# Correspondence to General Practitioners Following Clinic Appointments at an Old Age Psychiatry Service

**DOI:** 10.1192/bjo.2026.11547

**Published:** 2026-06-30

**Authors:** Juliana Campbell, Olakunle Ashaye

**Affiliations:** 1 Chershire and wirral NHS trust, Chershire, United Kingdom; 2 Hertfordshire Partnership foundation Trust, Hertfordshire, United Kingdom

## Abstract

**Aims:**

To ensure content of letters to GPs contain, diagnosis, treatment plan and staff involved

To see if letters are copied to patients

To see if clinic letters are sent out within a week

To see if GP letters are copied to patients

**Methods:**

A review of three months clinic letters at an old age psychiatry service north of London was carried out to see if the letters were sent out within a week, copied to patients and included relevant information like diagnosis and management plan. A proforma was designed to collect the relevant information.

**Results:**

In the period 1st May to 31 July, 163 clinic letters were sent out. The mean age of patients was 76.4 year and there were 96 (58.9%) female patients. In terms of diagnoses in the letters, 79 (48.5%) had dementia, 52 (31.9%) had depression and 14 (8.6%) had psychotic disorder (schizophrenia or delusional disorder). A management plan was included in 153 letters and 160 clinic letters were copied to patients or their carers.

11 of the 163 (6.7%) clinic letters were sent later than 7 days of clinic appointments

**Conclusion:**

Areas of Good Practice:

All clinic letters except three were copied to patients

Over 90% of clinic letters were sent within a week of clinic appointment

Areas of Improvement:

To achieve the desired goal of 100% of clinic letters being sent out within a week.

It is expected with improving information technology, letters will be uploaded onto patients’ primary care records electronically and become immediately accessible to both patients and general practitioners once sent. This is already the case in many practices locally and will give a more accurate picture of when clinic letters are received. The disadvantage will be for those patients and carers who do not have internet access or IT skills to access their primary care records

